# Autophagy manages disease-associated stress granules

**DOI:** 10.18632/oncotarget.5902

**Published:** 2015-09-28

**Authors:** Jin-A Lee

**Affiliations:** Department of Biological Science and Biotechnology, Hannam University, Daejeon, Korea

**Keywords:** FUS, FTD, ALS, autophagy, stress granule

Abnormal accumulation of cytosolic aggregates or inclusion bodies is a hallmark of several neurodegenerative diseases. Many common or specific molecular components such as ubiquitin, α-synuclein, TDP-43, or tau in neuronal cytosolic inclusion bodies have been identified as being associated with cellular pathogenic mechanisms of disease. Recently, stress granule (SG) marker proteins such as PABP and G3BP were found in cytosolic inclusion bodies of patients with frontotemporal lobar dementia (FTLD), alzheimer's disease (AD), or amyotrophic lateral sclerosis (ALS), suggesting that inappropriate SG dynamics contribute to pathogenic mechanisms [1]. Moreover, disease-associated tau, ataxin-2, survival of motor neurons (SMN), and angiogenin (ANG) proteins are recruited to SGs under stress conditions, and mutations in FMRP or hnRNPA1 are known to cause severe impairment of SG dynamics. Collectively, these facts suggest a common possible role for SG dysregulation in neurodegeneration. In detail, SGs are dense aggregates composed of RNA-binding proteins and non-translating mRNAs that are rapidly assembled upon stress exposure to inhibit improper mRNA translation and cell death and then disassembled thereafter [2]. Therefore, fine regulation of SG dynamics and homeostasis is very important for the stress response and cell survival, especially in non-dividing cells such as neurons. Despite prior studies on dynamics of SGs, regulatory mechanisms of disease-associated SGs in the context of neuronal degradation as well as the roles of persistent SGs in neurodegeneration are largely unknown.

To address these questions, our study investigated the role of autophagy, which controls the quality of proteins or organelles, in the regulation of ALS-linked SGs and neurodegeneration in disease-associated cultured neurons [3]. Fused in sarcoma (FUS), which was initially identified as a fusion oncogene, is a multifunctional RNA-binding protein whose mutations have been detected in ALS and FTLD [4]. In our study, ALS-linked FUS (R521C) mutation caused persistent SG accumulation upon oxidative stress, likely due to impairment of FUS disassembly from SGs, which is consistent with other cellular studies on *FUS* mutations [5]. Interestingly, mutant FUS-positive SGs showed greater accumulation in autophagosomes due to reduced autophagic flux compared to wild-type FUS-positive SGs, thereby supporting an active role for autophagy in regulation of SGs in neurons. Moreover, we directly confirmed autophagic regulation of physiological SGs in neurons based on our observation that inhibition of autophagy using *atg7* siRNA increased the number of SGs, whereas activation of autophagy reduced SG accumulation. Further analysis showed that mutant FUS-positive SGs were regulated by autophagy regulation in post-mitotic neurons. Most importantly, autophagy activation reduced mutant FUS-positive SG accumulation as well as mutant FUS-mediated neurite fragmentation and cell death in response to oxidative stress in disease-associated cultured neurons, suggesting a role for persistent SGs in neurodegeneration as well as involvement of autophagy in ALS-linked SG regulation. However, this finding needs to be confirmed in transgenic animal models and patients with specific iPSC-derived neurons associated with persistent SGs and SG aggregates [6].

Our study provides important insights into the roles of autophagy and disease-associated SGs in neurodegeneration. SGs are generally considered as a defense mechanism in neurons under stress conditions. However, persistent SGs in neurons might actually inhibit protein translation by sequestering mRNA and RNA-binding proteins, which are normally involved in transcriptional regulation in the nucleus or transport of mRNAs into dendrites/axons. Therefore, persistent SGs can lead to accumulation of toxic components/loss of essential components even after stress. According to our unpublished data, mutant FUS sequesters mRNAs that are involved in neuronal integrity into persistent SGs, which induces neurite fragmentation/shrinkage caused by oxidative stress in cortical neurons.

Moreover, our studies have shown that autophagy deficiency in *atg5−/−* MEFs causes SG formation/accumulation without any stress, suggesting that cellular homeostasis of SGs is tightly associated with autophagy. During normal aging or disease progression in the brain, dysregulated or insufficient autophagy combined with environmental factors such as acute or chronic stresses may accelerate SG formation and development of persistent SGs into larger cytosolic inclusions, leading to increased stress vulnerability in neurons as well as neurodegeneration.

However, the following questions remain to be answered in the context of autophagic regulation of SGs in physiology and pathology: How do cells regulate and harmonize formation/disassembly/autophagic degradation of SGs in response to different stresses in physiology and pathology? Which signals induce autophagic clearance of physiological or pathological SGs as opposed to disassembly of SGs or fusion with P-bodies? How are transient or persistent SGs recognized by autophagy machineries? What is the specific role of autophagy in regulation of SGs in relation to neuronal RNA granules such as P-bodies or transporting RNA granules in polarized neurons? More detailed studies are needed to elucidate autophagic regulation of SGs. Therefore, future efforts will provide novel opportunities for therapeutic intervention by targeting regulation of SGs via manipulation of the autophagic pathway in several neurodegenerative diseases associated with SGs such as ALS [7].
